# Adsorption Properties and Mechanisms of Methylene Blue by Modified Sphagnum Moss Bio-Based Adsorbents

**DOI:** 10.3390/ma17174329

**Published:** 2024-08-31

**Authors:** Junpeng Ren, Shijiang Zhang, Yu Wang, Hengxiu Yang

**Affiliations:** 1 School of Chemistry and Materials Science, Guizhou Education University, Guiyang 550018, China; rjunpeng@126.com (J.R.); 18212717435@163.com (S.Z.); 2 Guizhou Provincial Key Laboratory of Computational Nano-Material Science, Guizhou Education University, Guiyang 550018, China; hxyang@gznc.edu.cn

**Keywords:** sphagnum moss, modification, adsorption, methylene blue, adsorption mechanisms

## Abstract

The abundant pore structure and carbon composition of sphagnum peat moss render it a bio-based adsorbent for efficient methylene blue removal from wastewater. By utilizing sphagnum moss sourced from Guizhou, China, as raw material, a cost-effective and highly efficient bio-based adsorbent material was prepared through chemical modification. The structure and performance of the modified sphagnum moss were characterized using SEM, EDS, FTIR, and TGA techniques. Batch adsorption experiments explored the effects of contact time, adsorbent dosage, pH, initial dye concentration, and temperature on adsorption performance. Kinetics, isotherm models, and thermodynamics elucidated the adsorption behavior and mechanism. The modified sphagnum moss exhibited increased surface roughness and uniform surface modification, enhancing active site availability for improved adsorption. Experimental data aligned well with the Freundlich isotherm model and pseudo-second-order kinetic model, indicating efficient adsorption. The study elucidated the adsorption mechanism, laying a foundation for effective methylene blue removal. The utilization of modified sphagnum moss demonstrates significant potential in effectively removing MB from contaminated solutions due to its robust adsorption capability and efficient reusability.

## 1. Introduction

Water pollution is a grave environmental issue, posing significant threats to both human health and aquatic organisms as well as ecosystems. The presence of organic dye pollutants in wastewater has become a global concern, particularly originating from industries such as printing, textiles, cosmetics, and pharmaceuticals [1,2]. These pollutants are characterized by their complex composition, high toxicity levels, and resistance to biodegradation [3]. If left untreated before discharge into the environment, they can pose severe risks to both the ecosystem and human well-being. In fact, large quantities of organic pollutants are discharged into water bodies annually [4].

Organic synthetic dyes are extensively used due to their vibrant colors and light sensitivity [5]. However, the uncontrolled release of organic dye wastewater (e.g., methyl orange, malachite green, methylene blue, methyl red) from textile manufacturing processes or other related industries significantly contributes to pollution levels. Such untreated waste liquids not only disrupt the activity of aquatic organisms but also lead to cell carcinogenesis in individuals who consume contaminated water, ultimately inflicting damage upon essential organs like the liver, kidneys, and eyes, thus posing substantial risks to human well-being [6].

Notably, methylene blue (MB), a cationic dye favored for its chemical stability and color properties, finds application across multiple sectors, including textile, medical, and scientific research [7,8]. Despite its benefits, MB’s high solubility and toxicity classify it as a detrimental pollutant capable of ascending the food chain via direct contact or consumption, thereby endangering both human and animal health. Moreover, the chemical structure of methylene blue contains a benzene ring and methylene group, which confer high stability, making it resistant to natural degradation [9]. Therefore, effective treatment measures for dye-containing wastewater are crucial in preventing major health risks associated with environmental discharge. Consequently, studying efficient removal methods for dyes from wastewater holds great significance for environmental protection and resource recycling. Currently, predominant approaches for wastewater decolorization comprise adsorption [10,11,12], membrane filtration [13], photocatalytic degradation [14,15], advanced oxidation [16], etc., However, the drawbacks of these methods, such as non-biodegradability, high cost, and process complexity, have significantly limited their application in treating large-scale production wastewater. Conversely, adsorption emerges as a straightforward, cost-effective, and eco-friendly technique for water reclamation. This process entails the adhesion of ions or molecules from the aqueous phase onto the surface of an adsorbent material [17]. This selective attraction and retention make it an ideal choice for many industrial wastewater flows based on dye chemical properties. Bio-based adsorption materials have gained wide popularity in water pollution treatment due to their low cost, high removal efficiency, easy recovery/reusability of the adsorbent material without causing secondary pollution issues, and versatile applications. Several low-cost bio-based materials like coconut wastes [18], cactus [19], rice husk [20], luffa fiber [21], cotton husk bracts [7], sugar cane residue [22], betel nut shell [6], and corn straw [12] have been utilized to reduce dye concentrations and explore possibilities for water treatment purposes. Nevertheless, traditional adsorbents suffer from limitations, including low efficiency, high costs, poor reusability, and complex operations; hence, there is a pressing need to explore and develop new types of adsorption materials.

Sphagnum moss, a plant thriving in damp environments, primarily comprises cellulose, lignin, and other constituents, exhibiting a porous structure and a significant specific surface area [23]. Found in countries like Brazil, Ireland, China, and the United Kingdom, it is biocompatible, biodegradable, non-toxic, and cost-efficient. Sphagnum moss contains abundant oxygen-containing functional groups and aromatic structural substances, which provide numerous active sites for organic matter adsorption. Additionally, it exhibits a high cation exchange capacity, enabling the absorption of diverse water pollutants through ion exchange and complexation reactions [24]. While extensive research has been conducted on sphagnum moss-based adsorption materials for the effective removal of heavy metals like Cd, Pb, Cu, Ni, and Cr [25,26,27,28], there are limited studies on dye wastewater adsorption, particularly regarding methylene blue dye’s adsorption process and mechanism [29]. This paper utilizes sphagnum moss sourced from Guizhou Province in China as raw material to enhance its adsorption efficiency through appropriate chemical modification of organic compounds. The study systematically investigates the adsorption performance of methylene blue dye by conducting batch adsorption experiments while exploring various experimental parameters to determine their impact on the adsorption capacity. Furthermore, the study delves into the thermodynamics, kinetics, and isotherms of the adsorptive process to elucidate its mechanism, thereby providing novel ideas and methods for the efficient treatment of dye wastewater. This research represents the first detailed study in the literature on organic pollutant removal using Sphagnum moss.

## 2. Experimental Section

### 2.1. Materials

In this research, sphagnum moss (SM) collected from Guizhou Province, China, served as the adsorbent material. Before application, the moss underwent a pretreatment process involving sodium hydroxide (NaOH) and hydrogen peroxide (H_2_O_2_), sourced from Aladdin Reagent (Shanghai, China) Co., Ltd. Additional modifying agents such as silane, silicon dioxide, and sulfuric acid (H_2_SO_4_) and nitric acid (HNO_3_), were obtained from Sinopharm Beijing Co., Ltd. (Beijing, China). Ethanol, a chemically pure substance, was acquired from Tianjin Hengxing Chemical Co., Ltd. (Tianjin, China), while methylene blue and potassium bromide were supplied by Tianjin Kemeiou Chemical Reagent Co., Ltd. (Tianjin, China). All chemicals utilized in the study were of analytical grade and were used as received without further purification.

### 2.2. Preparation of the Materials

The sphagnum moss was crushed and dried in preparation of the materials. Subsequently, 10 g of this moss was added to a 1 L three-necked flask, combined with 800 mL of a sodium hydroxide solution at 0.0375 mol/L concentration, and the mixture was agitated. This blend was magnetically stirred at 95 °C within a water bath. Subsequently, hydrogen peroxide (2.0 mL, 30%) was incrementally introduced, with stirring continuing for an hour. After the reaction ended, the mixture was allowed to cool to ambient temperature, and its pH was balanced to neutral using a dilute sulfuric acid solution (0.1 mol/L). Vacuum filtration facilitated the separation of the treated sphagnum moss, which was then vacuum-dried at 50 °C for six hours.

The pretreated sphagnum moss (6 g) was placed in a beaker containing nitric acid (60 mL, 14.4 mol/L) and sulfuric acid (120 mL, 18.4 mol/L), maintaining a 1:2 volume ratio of nitric to sulfuric acid. The mixture was stirred for five minutes, then heated in a water bath at 31 °C for 20 min. Once the reaction was complete, the material was filtered and rinsed until a neutral pH was achieved in the filtrate. Subsequently, it was dried at 50 °C for 24 h to obtain the adsorption material of modified sphagnum moss.

### 2.3. Analytical Methods

Various analytical techniques were applied to assess the morphology and characteristics of sphagnum moss samples before and after modification. Surface morphology and structure were examined using scanning electron microscopy (SEM; Gemini 300, Zeiss Oberkochen, Germany), while electron diffraction scattering (EDS) analysis was conducted using Smart EDX, Zeiss, Germany. The infrared spectrum of sphagnum moss before and after modification was analyzed using an FT-IR spectrometer (Tensor 27, Bruker, Karlsruhe, Germany). The sample was mixed with potassium bromide in a mass ratio of 1:200 and pressed into a pellet. FT-IR spectra were collected in the 4000–400 cm^−1^ range for each sample. The thermal stability of the sphagnum moss was characterized using thermogravimetric analysis (TGA) (209 F1, Netzsch, Sable, Germany). The analysis took place in a nitrogen atmosphere with a heating rate of 10 °C·min^−1^, covering a temperature range from 25 °C to 800 °C.

### 2.4. Batch Adsorption Experiments

A stock solution (300 mg/L) was prepared by dissolving the appropriate amount of methylene blue (MB) in deionized water. Batch experiments were conducted to investigate the impact of contact time (0–210 min), pH of the dye solution (4–14), adsorbent dosage (0.3–2 g/L), initial dye concentration (20–200 mg/L), and temperature (298–328 K). In each experiment, except for one that aimed to test the effect of adsorbent dosage, 0.05 g of modified sphagnum moss was added to 30 mL of MB solution after adjusting its pH. The mixture was then agitated at a rate of 150 r/min for 180 min until reaching equilibrium conditions at room temperature. Separation of adsorbate from adsorbent occurred through sand core funnel filtration. The supernatant solution was collected by removing it from the filter bottle, and its residual concentrations of MB were measured using a UV–visible spectrophotometer to determine absorbance levels (UV-1800, Shimadzu, Kyoto, Japan; Specord210 plus, Analytik Jena AG, Jena, Germany). The residual concentration could be determined from the calibration curve using Beer–Lambert law principles. Each sample underwent three tests, and an average value was taken to ensure accuracy and repeatability. Removal efficiency R and adsorption capacity $q_{e}$ (mg/g) were determined using Equations (1) and (2), respectively,

(1) $$R=\frac{C_{0}-C_{e}}{C_{0}}\times 100\%$$

(2) $$q_{e}=\frac{V}{m}\left(c_{0}-c_{e}\right)$$

Initial MB concentration ($C_{0}$) in mg/L, equilibrium concentrations ($C_{e}$) in mg/L, batch solution volume (*V*) in L, and the adsorbent mass (*m*) in g were utilized.

### 2.5. Adsorption Isotherms

An amount of 0.05 g of the modified sphagnum moss was added to centrifuge tubes containing 30 mL of MB solutions at the optimal pH. The initial concentrations of the MB solutions were adjusted to 100, 120, 140, 160, 180, 200, and 220 mg/L. The centrifuge tubes were placed in a temperature-controlled water bath shaking box and agitated for a duration of 12 h at a constant temperature of 298 K and a speed of rotation at 150 r/min. The adsorption processes followed the method described in Section 2.4 above. Subsequently, the temperature was adjusted to both 308 K and 318 K for further repetitions of the aforementioned procedure. The experimental data were fitted using both Langmuir and Freundlich models to determine the relevant parameters associated with these adsorption isotherm models [7,12]. The formulas were presented by Equations (3) and (4):

Langmuir:

(3) $$\frac{c_{e}}{q_{e}}=\frac{c_{e}}{q_{m}}+\frac{1}{K_{L}q_{m}}$$

Freundlich:

(4) $$\ln q_{e}=\ln K_{F}+\frac{1}{n}\ln c_{e}$$

The equilibrium adsorption capacity ($q_{e}$) is represented in mg/g, while $q_{m}$ represents the saturation adsorption capacity in mg/g, $C_{e}$ represents the concentration of MB in the solution at adsorption equilibrium (mg/L). $K_{L}$ denotes the Langmuir constant, which relates to the adsorption-free energy and affinity of adsorption sites. The Freundlich constants, $K_{F}$ and *n*, represent the system’s adsorption capacity and strength, respectively. By linearly fitting ($\frac{c_{e}}{q_{e}}$) against ($c_{e}$), $K_{L}$ and $q_{m}$ can be determined from the slope and intercept of the fitted line, respectively. For favorable adsorption, the Freundlich constant should fall within a range of 1–10. These parameters can be obtained from plotting $\ln q_{e}$ vs. $\ln C_{e}$.

### 2.6. Adsorption Kinetics

In this experiment, the modified sphagnum moss, weighing 0.05 g, was individually placed into 100 mL conical flasks containing 30 mL of methylene blue (MB) solution with varying initial concentrations of 150 mg/L and 200 mg/L at the optimal pH and temperature of 298 K. Subsequently, the flasks underwent adsorption experiments at room temperature in a shaking water bath. The time-dependent adsorption capacity of the modified sphagnum moss was monitored using a UV–visible spectrophotometer, and kinetic mechanisms were analyzed employing various models (Equations (5)–(7)) [13,30].

Pseudo-first-order kinetic model:

(5) $$\ln(q_{e}-q_{t})=\ln q_{e}-k_{1}t$$

Pseudo-second-order kinetic model:

(6) $$\frac{t}{q_{t}}=\frac{1}{k_{2}q_{e}^{2}}+\frac{t}{q_{e}}$$

Intra-particle diffusion model:

(7) $$q_{t}=k_{id}t^{\frac{1}{2}}+C$$

The adsorption time (*t*) is expressed in minutes, while the equilibrium adsorption amount ($q_{e}$) and the amount adsorbed at time *t* ($q_{t}$) are measured in mg/g. The rate constants for the pseudo-first-order model ($k_{1}$) and pseudo-second-order model ($k_{2}$) are also denoted. Additionally, *C* represents a constant parameter associated with the thickness of the boundary layer, while $k_{id}$ corresponds to a constant parameter related to the intra-particle diffusion rate constant.

### 2.7. Thermodynamics

The thermodynamic adsorption isotherm experiments elucidate the variation in Gibbs free energy of the adsorbent throughout the wastewater adsorption process. The alteration in free energy can be determined utilizing the subsequent two equations: [6,31].

(8) $$K_{D}=\frac{q_{e}}{c_{e}}$$

(9) $$\Delta G^{0}=-RT\ln K_{D}$$

The change in enthalpy ΔH^0^ and the change in entropy ΔS^0^ can be calculated based on the aforementioned equations. The values of ΔH^0^ and ΔS^0^ can be determined by analyzing the intercept and slope of the $\ln K_{D}$ versus $\frac{1}{T}$ plot.

(10) $$\ln K_{D}=\frac{1}{R}(\Delta S^{0}-\frac{{\Delta H}^{0}}{T})$$

### 2.8. Adsorption Regeneration Experiment

The regenerative performance was demonstrated by conducting adsorption–desorption cycles using 0.05 g of modified sphagnum moss in a 30 mL solution containing 200 mg/L of MB at the optimal pH and temperature of 298 K for the adsorption experiments. Stirring occurred at 150 r/min for 3 h at room temperature. Subsequently, the modified sphagnum moss was filtered, dried, and subjected to desorption with absolute ethanol for 2 h to extract MB from the sample. Following desorption, the obtained modified sphagnum moss was washed and dried for reuse in subsequent rounds of adsorption experiments. This cycle was repeated five times, with the removal rate of MB by the sphagnum moss adsorbent in each experiment serving as an indicator to evaluate its regeneration performance.

## 3. Result and Discussion

### 3.1. Analyses of Morphologies

**Figure 1:**
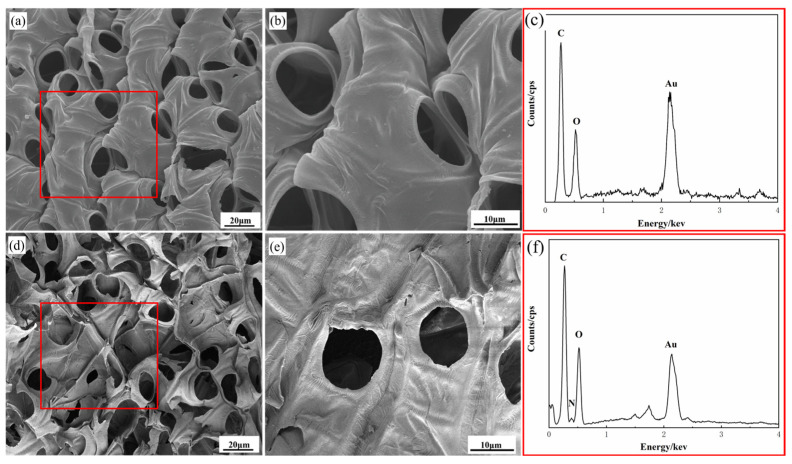
SEM images of sphagnum moss before and after modification: (**a**,**b**) original sphagnum moss; (**c**) EDS analysis of original sphagnum moss corresponding to the red frame labeled in (**a**); (**d**,**e**) sphagnum moss after modification; (**f**) EDS analysis of modified sphagnum moss corresponding to the red frame labeled in (**d**).

**Figure 2:**
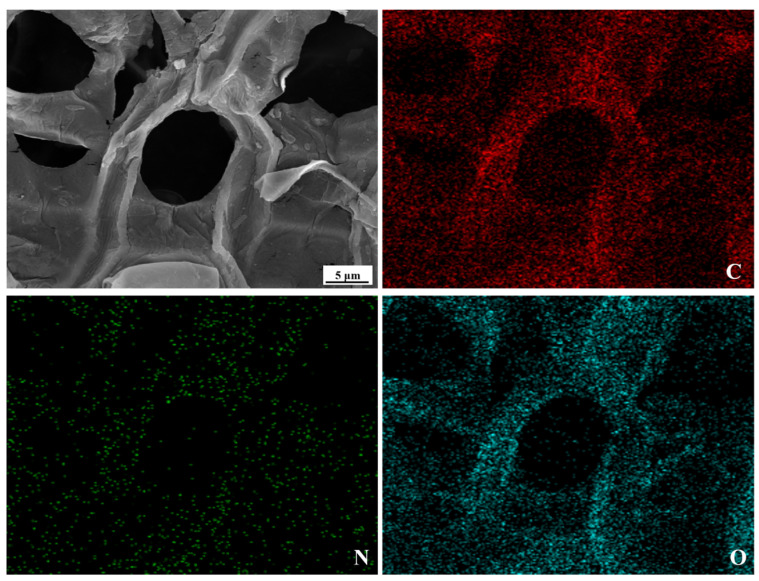
The SEM image of sphagnum moss through modification and its corresponding elemental mapping images of C, O, and N.

The surface morphologies of both raw and modified sphagnum moss are depicted in Figure 1. As observed from the original morphology of sphagnum moss (Figure 1a,b), the material’s surface is abundantly porous, displaying a smooth and pristine appearance that is highly suitable for adsorption material preparation [29]. Upon comparing the raw material with its modified counterpart, it becomes evident that the surface of the modified sphagnum moss exhibits increased coarseness and blurred edges, forming a porous network structure that offers numerous active sites for subsequent grafting (Figure 1d,e). Moreover, the analysis utilizing the EDS spectrum has revealed the co-existence of N elements alongside C and O elements on the surface of the modified sphagnum moss, indicating a modification in its surface composition (Figure 1c,f). This modification likely improves the interaction between the modified sphagnum moss and the dye, potentially boosting the adsorption capacity. Analyzing both the SEM image of the modified sphagnum moss and its corresponding elemental mapping images of C, O, and N presented in Figure 2 allows us to conclude that uniform surface modification reactions using concentrated sulfuric acid and concentrated nitric acid have occurred on the surface of sphagnum moss.

### 3.2. Infrared Spectroscopic Analysis

**Figure 3:**
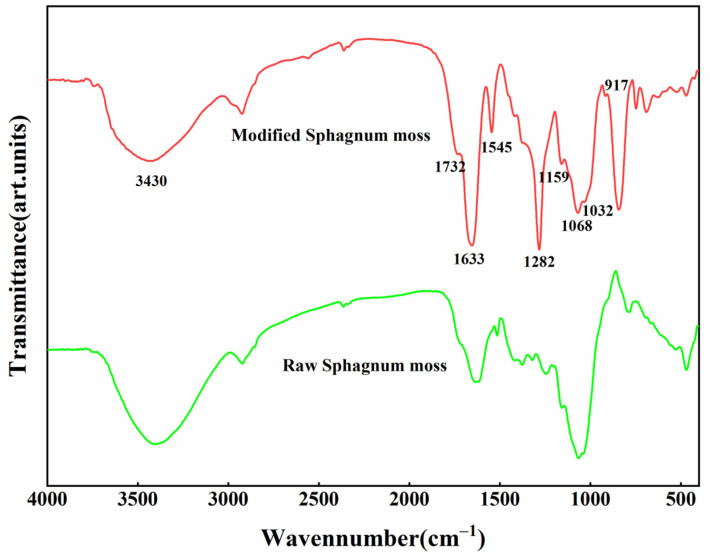
Infrared spectra of sphagnum moss before and after modification.

In order to assess the changes in surface information of samples before and after modification, infrared spectrometry was employed to analyze the functional groups present in the materials. Figure 3 presents the FT-IR spectra of both raw and modified sphagnum moss within the 400–4000 cm^−1^ range.

Within the high-frequency region, a prominent peak at 3430 cm^−1^ was observed in raw and modified sphagnum moss samples, indicating the presence of hydroxyl (-OH) groups. However, the adsorption peak corresponding to -OH on modified sphagnum moss exhibited significant attenuation, suggesting a reaction with HNO_3_ and substitution by nitro groups (-NO_2_). The introduction of nitrogen-containing functional groups onto the surface of sphagnum moss through nitric acid functionalization is confirmed by observing a characteristic nitro (-NO_2_) peak at 1545 cm^−1^ [32]. The characteristic peak for aromatic -C=C- bonds is observed at 1032 cm^−1^, while bands within the range of 1650–1600 cm^−1^ correspond to carbon-based carboxyl bonds [24]; additionally, a distinctive peak formed by polysaccharide carbonyl can be seen at 1633 cm^−1^. Due to the disruption of the internal structure during the modification process, chemical bonding is altered, leading to changes in the types of surface functional groups present. At 1732 cm^−1^, there exists a carbon-based bond associated with carboxylic acid compound (-COOH) and ester functionality, an absorption peak related to stretching vibration for -C=C- occurs at 1418 cm^−1^ [12]; furthermore, a strong absorption peak attributed to contraction vibration for -C-O- appears at 1282 cm^−1^. Simultaneously, a new characteristic peak emerges between 1300 and 1050 cm^−1^, which corresponds to stretching vibrations for ester’s -C-O- group, and ether’s C-O-C group stretching vibrations are also evident within this range [8,33]. Lastly, the adsorption peak detected at a wavenumber value of 917 cm^−1^ arises from C-H bending vibration.

### 3.3. Thermogravimetric Analysis

**Figure 4:**
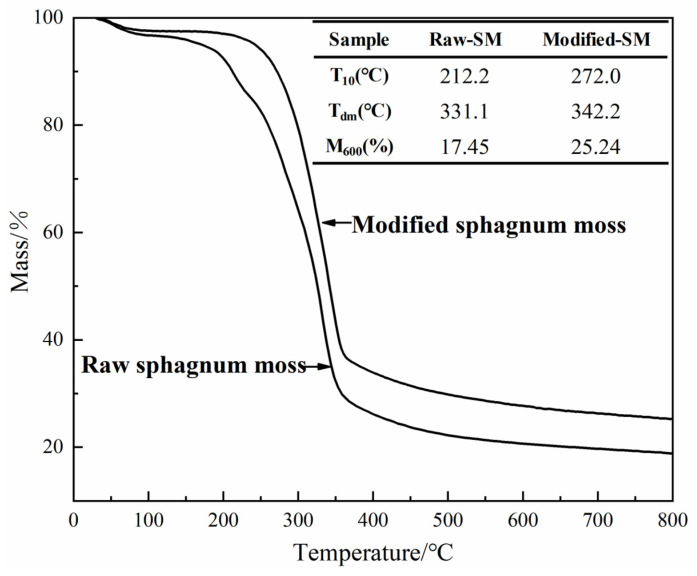
Thermogravimetric analysis of raw sphagnum moss and modified sphagnum moss.

The thermal stability of the sphagnum moss before and after modification was confirmed through thermal weight analyses, as depicted in Figure 4. TGA curves exhibited slight mass loss below 200 °C, which was attributed to continuous volatilization of water, hydrocarbons, and other volatile organic compounds [19,34]. The sphagnum moss material primarily comprises plant-based cellulose and lignin tissue, with a pyrolysis temperature range of 300–373 °C [35].

Evaluation of thermal stability involved comparing temperatures T_10_ (10% mass loss) and Tdm (maximum decomposition rate) before and after modification. The surface modification increased T_10_ from 212.2 °C to 272.0 °C and T_dm_ from 331.1 °C to 342.2 °C, indicating enhanced thermal stability. At a temperature of 600 °C, there was minimal change in mass with some residual sample remaining. The modified adsorbents exhibited a maximum weight loss temperature range between 270 and 340 °C, demonstrating their stability within operational temperatures.

### 3.4. Adsorption Experiments

#### 3.4.1. The Influence of Contact Time and Initial MB Concentration on Adsorption Effect

**Figure 5:**
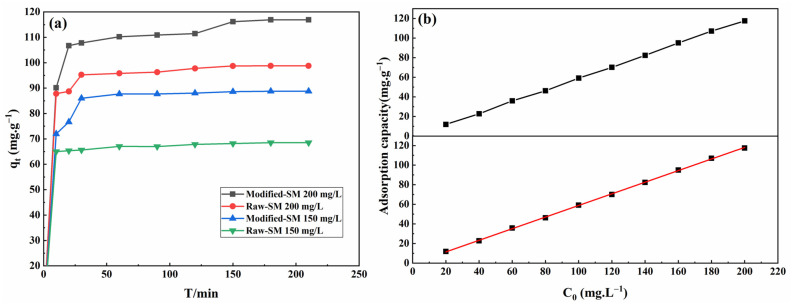
The effect of contact time and initial MB concentration on the adsorption capacity of sphagnum moss before and after modification: (**a**) the curve of adsorption capacity over time; (**b**) the effect of MB concentration on adsorption capacity corresponding to the grey line and the influence of MB concentration on adsorption capacity were linearly fitted corresponding to the red line.

To examine the effects of contact duration and starting concentration on the adsorption capabilities of sphagnum moss, experiments were conducted using methylene blue (MB) solutions, with results displayed in Figure 5. Figure 5a shows a comparison of adsorption capacities between untreated and chemically modified sphagnum moss at initial MB concentrations of 150 mg/L and 200 mg/L. The modified sphagnum moss exhibited significantly enhanced adsorption efficiency compared to the original sphagnum moss, with an increase in adsorption capacity from 68.528 mg/g to 88.767 mg/g and from 98.775 mg/g to 116.898 mg/g for the respective concentrations. Moreover, the adsorption efficiency improved from 76.1% and 82.31% to 98.63% and 97.41%, respectively. Furthermore, it can be observed that there is a rapid increase in MB uptake by the modified sphagnum moss material within the first 35 min of contact time, followed by a gradual approach towards equilibrium without any substantial rise thereafter. This swift initial adsorption is attributed to the large surface area of the moss, the strong interaction between MB molecules and sphagnum moss surface, and an abundance of accessible active sites on the modified moss [36]. The overall process of MB adsorption onto modified sphagnum moss can be divided into two distinct stages. In the first stage, there is evident electrostatic adsorption between MB solution and active sites on the adsorbent’s surface, with the reaction primarily occurring at the surface. The second stage involves diffusion of the MB solution absorbed on the surface into the pores of the adsorbent. As a result of significant internal diffusion resistance, as adsorption progresses, saturation gradually occurs at active sites while reducing concentration differences between MB inside and outside the adsorbent until reaching equilibrium.

Figure 5b illustrates a proportional rise in the adsorption capacity of modified sphagnum moss across a range of MB concentrations from 20 mg/L to 200 mg/L, suggesting a strong linear correlation. The observed findings can be attributed to the interaction between the MB solution and the active sites of modified sphagnum moss. A higher initial concentration of MB enhances the driving force for migration towards these active adsorption sites [37], thereby increasing collision probability between MB molecules and modified sphagnum moss adsorption sites. Consequently, this promotes greater entry of methylene blue molecules into unoccupied adsorbent pores. Therefore, increasing the initial concentration of MB potentially boosts the adsorption efficiency of MB wastewater treatment.

#### 3.4.2. The Influence of the MB Solution pH on Adsorption Effect

**Figure 6:**
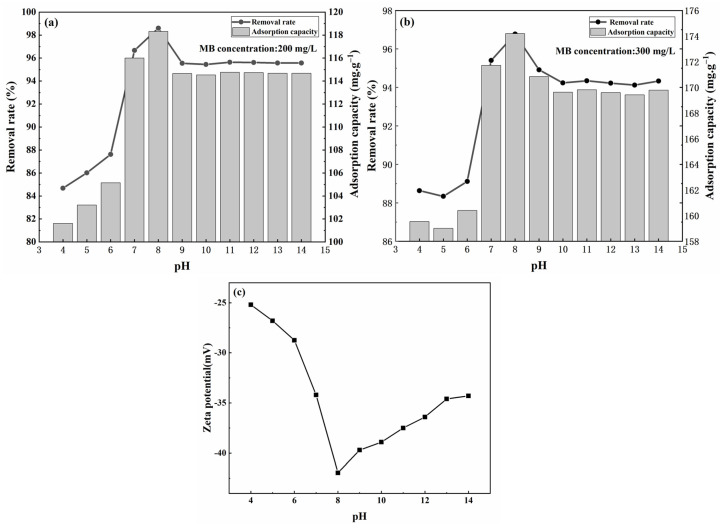
Effect of sphagnum moss after modification on MB adsorption under different pH: (**a**) initial MB concentration of 200 mg/L; (**b**) initial MB concentration of 300 mg/L; (**c**) zeta potential change in modified sphagnum moss with pH.

The impact of pH on the adsorption performance of modified sphagnum moss at varying concentrations of MB is depicted in Figure 6a,b. The adsorbent’s efficacy in removing MB is contingent upon the pH values. The removal efficiency of MB exhibits an ascending trend up to pH 8, followed by a decline beyond this point.

The reasons for this phenomenon are as follows: As can be seen from Figure 6c, different acid–base environments will have a different impact on the surface charge of the adsorbent. Zeta potential measurements clearly indicate that the surface-modified sphagnum moss adsorbent maintains a negative zeta potential across a broad pH range of 4–14, facilitating strong electrostatic attraction with methylene blue molecules and resulting in relatively superior adsorption performance under various pH conditions [30]. However, at low pH, the negative surface charge of the adsorbent decreases, leading to reduced adsorption efficiency. This is due to competition between H^+^ ions and methylene blue molecules in acidic solutions, causing partial protonation of the adsorbent and a reduction in its surface negative charge [6,8]. Conversely, as the pH increases, the adsorbent undergoes deprotonation, which increases the negative surface charge. This enhancement in negative charge increases the number of adsorption sites on the adsorbent, thereby improving adsorption efficiency. However, when pH exceeds 8, deprotonation occurs, causing methylene blue to gradually transition from a positive to a negative charge state, which leads to a decline in the electrostatic adsorption mechanism and a subsequent decrease in the overall performance of dye wastewater removal by this system [38,39]. Therefore, optimizing the pH of the solution is crucial for maximizing MB adsorption onto modified sphagnum moss, as it directly influences the availability and accessibility of surface binding sites on the adsorbent.

#### 3.4.3. The Influence of Adsorbent Dosage on Adsorption Effect

**Figure 7:**
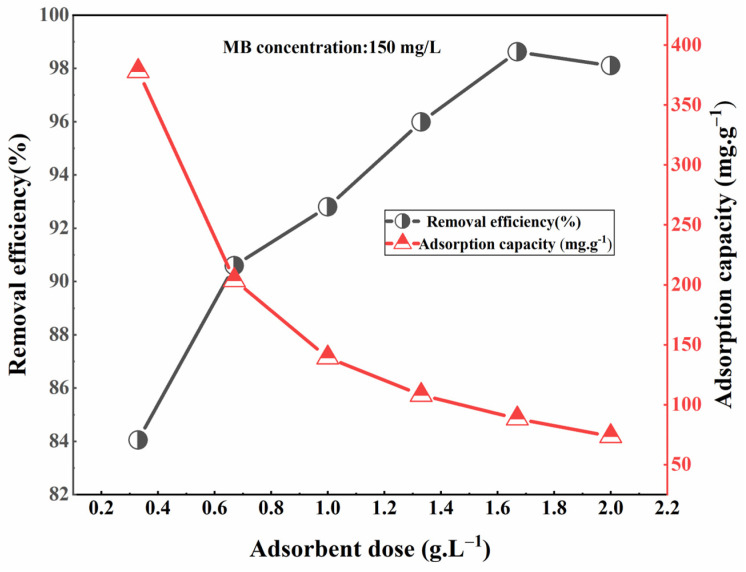
Effect of adsorbent dose on MB adsorption.

The results depicted in Figure 7 demonstrate the MB removal efficiency and adsorption capacity of modified sphagnum moss at different dosages. In this investigation, the removal efficiency exhibited an increase from 84.05% to 98.63% as the dosage of adsorbent was elevated from 0.3 to 2 g/L. This can be attributed to the augmentation of surface binding sites per unit volume with higher doses of adsorption. Nevertheless, there was a notable decline in adsorption capacity, which decreased from 378.227 to 73.53 mg/g.

The trend observed suggests that at lower doses, the surface adsorption groups on modified sphagnum moss effectively interacted with MB in solution, efficiently occupying active sites and enhancing the adsorbent’s capacity per unit mass. However, with an increase in the adsorbent mass, there was a decrease in contact between each unit mass of modified sphagnum moss and MB dye in solution, and the adsorption active point of the modified sphagnum moss did not reach saturation, which brought down the utilization of adsorption sites, lowering the adsorbed amount [12]. Additionally, excessive suspended adsorbents may have formed aggregates [31], resulting in a decreased specific surface area that hindered complete contact with MB dye and consequently impacted overall performance. In line with our objective to enhance both adsorption efficiency and cost-effectiveness while reducing expenses, an optimal dosage value for modified sphagnum moss was determined as 1.67 g/L through experimental analysis.

#### 3.4.4. Adsorption Isotherms

**Figure 8:**
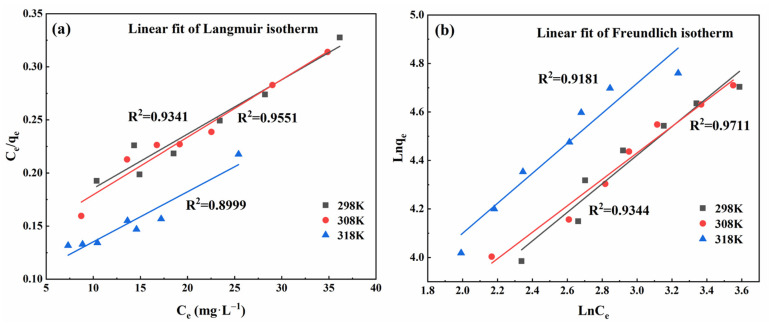
Langmuir model and Freundlich model of isothermal adsorption under different concentrations: (**a**) Linear fit of Langmuir isotherm; (**b**) Linear fit of Freundlich isotherm.

Adsorption isotherms are vital for the analysis and understanding of the thermodynamic parameters associated with adsorption, offering insights into the chemical interactions and mechanisms between pollutant molecules and the adsorbent surfaces [6,40]. A series of experiments utilizing both the Langmuir and Freundlich isotherm models was conducted to explore the adsorption behavior of modified sphagnum moss on MB (refer to Figure 8 and Table 1). The Langmuir model is applied to explain monolayer adsorption on homogeneous surfaces, whereas the Freundlich model addresses multilayer adsorption on heterogeneous surfaces. These experiments facilitate a deeper comprehension of the adsorption phenomena.

From the data presented in Table 1, it is evident that the R^2^ value for the Freundlich model surpasses that of the Langmuir model, indicating a superior ability of the Freundlich model to describe the adsorption process of modified sphagnum moss adsorbent on MB solution. Moreover, this suggests that multilayer adsorption predominantly occurs [11]. By plotting $\ln C_{e}$ against $\ln Q_{e}$, a linear Freundlich adsorption isotherm (Figure 8b) was observed. The slope and intercept of this equation provide insights into the parameters $\frac{1}{n}$ and *K_F_*, respectively. A $\frac{1}{n}$ value less than 1 indicates surface non-uniformity of the adsorbent, which enhances adsorption efficacy and promotes the adsorption process.

#### 3.4.5. Adsorption Kinetics

**Figure 9:**
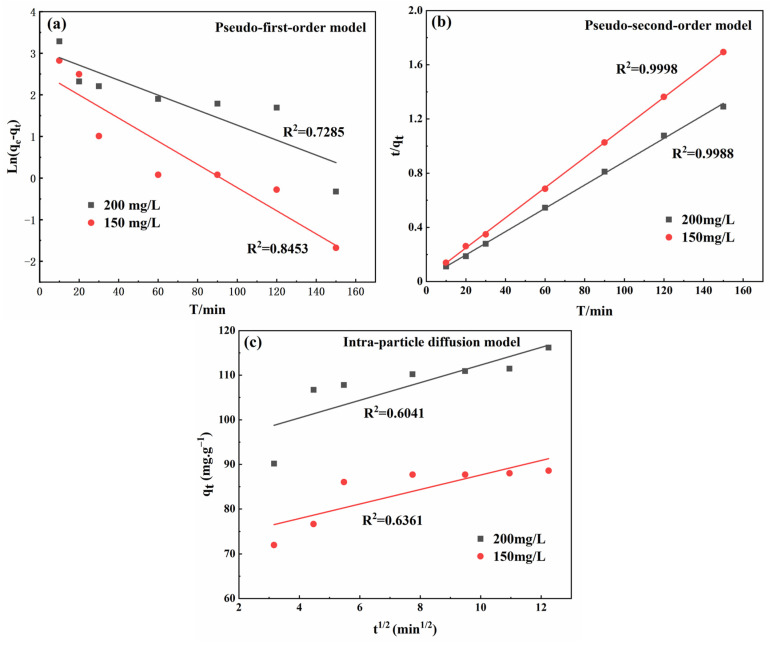
Adsorption kinetics of MB adsorbed by modified sphagnum moss: (**a**) pseudo-first-order kinetics model; (**b**) pseudo-second-order kinetics model; (**c**) intra-particle diffusion model.

The investigation of adsorption kinetics can offer a more comprehensive depiction of the rate at which adsorption occurs and establish a reasonable understanding of the adsorption mechanism. To investigate the kinetic mechanism of MB adsorption by surface-modified sphagnum moss adsorbent, various kinetic models, including pseudo-first-order, pseudo-second-order, and intra-particle diffusion models, were employed. Figure 9a–c illustrates the fitting results from these models, while Table 2 lists the corresponding parameters.

The superior performance of the pseudo-second-order kinetics, as evidenced by higher correlation coefficient (R^2^) values and a close match between calculated and experimentally observed equilibrium adsorption capacities (Figure 9a,b; Table 2), indicates that the MB adsorption process conforms to this model. This suggests a chemisorption process involving electron transfer between adsorbents and adsorbates [41]. Notably, at low concentrations, k_2_ surpasses its counterpart at high concentrations, which explains the rapid uptake observed at lower concentrations. The parameter k_2_: 0 < k_2_ < 1 signifies favorable absorption. Furthermore, according to the intra-particle diffusion model, intraparticle diffusion significantly influences the reaction rate. Based on the fitting results shown in Figure 9c, it is evident that there is a relatively low R^2^ value and poor linearity in the fitting for internal diffusion model analysis, indicating the minimal impact of intraparticle diffusion factors on MB adsorption by the adsorbent. Thus, intra-particle diffusion does not significantly impact the overall adsorption process.

#### 3.4.6. Adsorption Thermodynamics

**Figure 10:**
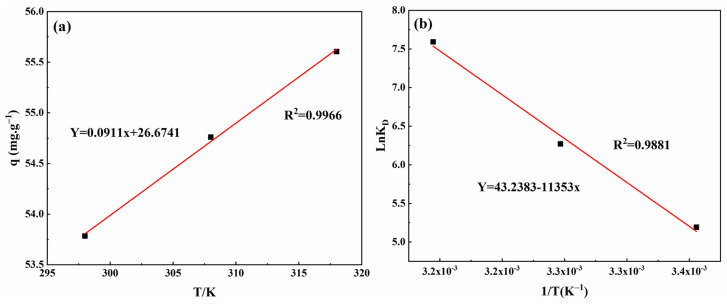
Thermodynamics fitting of modified sphagnum moss: (**a**) adsorption capacity temperture diagram; (**b**) $\ln K_{D}$ and temperature reciprocal linear fitting curve.

The thermodynamic parameters of adsorption—change in enthalpy (ΔH⁰), Gibbs free energy (ΔG⁰), and entropy (ΔS⁰)—are crucial in analyzing the adsorption process. These parameters determine the driving force and direction of the adsorption process, as well as elucidate its microscopic mechanism. By plotting the relationship between $\ln K_{D}$ and $\frac{1}{T}$, ΔH⁰ and ΔS⁰ were derived from the line’s slope and intercept, respectively (refer to Figure 10). Additionally, ΔG⁰ was computed using Equation (9), and the resulting values are detailed in Table 3.

According to Figure 10 and Table 3, an increase in temperature correlates with an enhanced adsorption capacity of the modified sphagnum moss adsorbent for MB. The positive ΔH⁰ suggests that the adsorption is endothermic, while a positive ΔS⁰ indicates irreversible adsorption and increased disorder at the interface between the adsorbent and MB. These findings imply that the adsorption is chemically driven, necessitating heat for effective dye uptake and promoting rapid diffusion to the adsorbent’s surface [8,42]. A negative value for ΔG⁰ indicates that external conditions are not necessary to promote the spontaneous feasibility of this absorption reaction. Additionally, it is observed that higher temperatures lead to decreased values of ΔG⁰, implying greater spontaneity within this absorption process. Furthermore, an increase in temperature can improve the adsorption performance of dyes.

#### 3.4.7. Adsorption–Desorption Cycle Experiments

**Figure 11:**
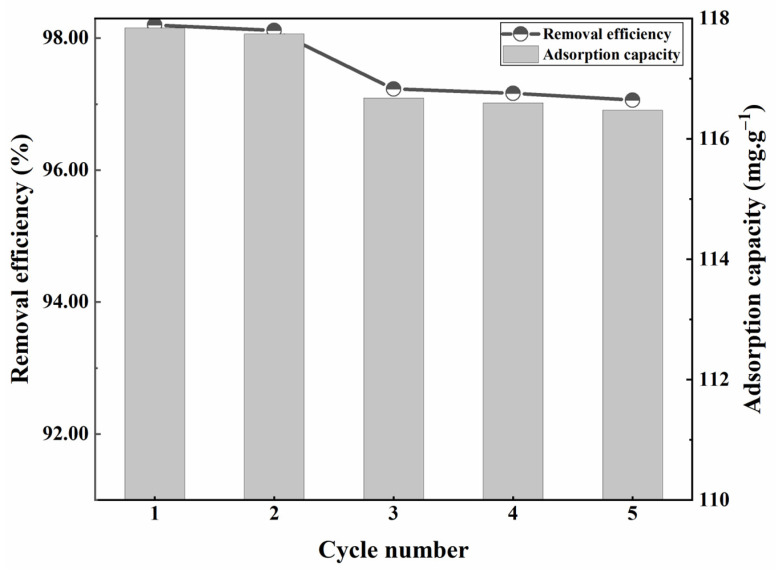
Adsorption–desorption regeneration experiment results of modified sphagnum moss for MB solution.

Reusability is a critical parameter for assessing the feasibility and stability of adsorbent materials. The results presented in Figure 11 demonstrate the performance of modified sphagnum moss adsorbents during five cycles of adsorption–desorption experiments. Following five cycles, there was only a marginal decrease in the adsorption efficiency, with the removal rate decreasing from 98.1% to 97%. These results indicate that the efficiency of adsorption remained within a reasonable range. After undergoing surface modification, the hydrophobicity of sphagnum moss was enhanced. During the removal of MB, the cationic organic molecule selectively enters the hydrophobic cavity of sphagnum moss to form the inclusion complex and facilitate rapid adsorption of MB molecules [43]. However, a slight reduction in effectiveness may be ascribed to hindrance from intermediate adsorbed molecules obstructing active sites and impeding the adsorption process of methylene blue dye. Additionally, the addition of ethanol as a desorption agent enhanced the hydrophilicity of the material and disrupted its hydrophobic cavities, consequently diminishing the adsorbent’s affinity for methylene blue wastewater and thereby influencing its adsorption performance [6,12,44]. Consequently, this leads to decreased affinity between the adsorbent material and MB wastewater, thereby reducing overall performance. Nevertheless, based on these cyclic experiments, it can be concluded that the modified sphagnum moss adsorbent demonstrates robust stability and noteworthy regenerative capability in MB adsorption.

### 3.5. Adsorption Mechanism

**Figure 12:**
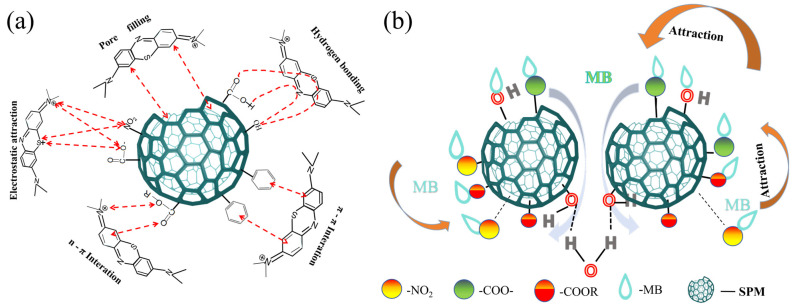
Mechanism of chemically modified sphagnum moss. (**a**) Mechanism of modified sphagnum moss for MB; (**b**) Diagram of the rapid adsorption of MB by modified sphagnum moss.

The adsorption mechanism involves interactions between the adsorbate molecules and the active sites on the adsorbent’s surface. Sphagnum peat moss, with its abundant cellulose, hemicellulose, lignin, and other components, holds great promise as a bio-based material. Biomass materials generally exhibit effective adsorption onto adsorbents through electrostatic interactions, hydrogen bonding, n-π and π-π interactions, as well as van der Waals forces [45]. The exceptional adsorption performance of modified sphagnum moss adsorbents can be attributed to several factors: Firstly, the surfaces of sphagnum moss possess numerous pores that facilitate MB wastewater absorption by filling these pores. However, due to limited surface adsorption sites, there are restrictions on its capacity for MB wastewater. By roughening the surface of modified sphagnum moss and increasing micropore numbers simultaneously, the penetration of methylene blue molecules into the absorbent material is enhanced via pore-filling effects, thereby promoting contact with modified surface adsorption sites and improving overall adsorption efficiency [31,46]. Secondly, hydrogen bonding plays a significant role in influencing the adsorption performance. Following surface modification, the surface structure of the modified sphagnum moss undergoes alterations. Apart from the successfully attached nitro group, there are also functional groups containing oxygen, including carboxyl and hydroxyl groups, present on the surface. During the process of MB adsorption, facile formation of hydrogen bonds occurs with nitrogen-containing functional groups present in methylene blue molecules, thereby enhancing the adsorption performance of the modified sphagnum moss [19,30,47]. Simultaneously, the introduction of nitro groups (-NO_2_) enhances the hydrophobicity of the material while effectively blocking water molecule adsorption, thereby improving the adsorption efficiency of methylene blue dye. On the other hand, the hydrophilic component in the material, like unreacted -OH on the surface of modified sphagnum moss, provided a possibility for the formation of hydrogen bonds among adsorbent particles as well as with water molecules (Figure 12b). Thus, agglomeration between adsorbent particles can be achieved after adsorption, which enables material recycling. Thirdly, robust electrostatic interactions occur between the modified sphagnum moss adsorbent and MB molecules. The modified adsorbent surface carries a substantial negative charge, facilitating robust electrostatic interactions with methyl blue (MB) molecules that are positively charged. This enhances their affinity for binding to the modified sphagnum moss. Furthermore, the surface of the modified sphagnum moss adsorbents possesses various functional groups, such as -NO_2_ and -COOH, which act as active sites for binding functional groups like S^+^ or N^+^ in the MB dye [6,7,12]. Fourthly, the presence of strong π-π conjugation due to the characteristic aromatic ring structure of the modified sphagnum moss contributes significantly to improved adsorption performance [48,49]. The carbon-carbon double bond on the modified sphagnum moss adsorbent interacts with the benzene ring of MB through π-π interactions, thereby promoting efficient adsorption. Lastly, it is worth noting the potential role of n-π interactions, where carbon-based oxygen in ester derivatives acts as an electron donor and the dye’s aromatic ring acts as an electron acceptor, may also play a significant role in enhancing MB adsorption.

## 4. Conclusions

The bio-adsorbent was prepared by surface modification of sphagnum moss sourced from Guizhou Province, China, using concentrated sulfuric acid and concentrated nitric acid. Its adsorption capacity for methylene blue was investigated. The modified sphagnum moss exhibited a rough surface structure with porous and irregular textures due to the replacement of hydroxyl groups by nitro groups, resulting in an increased number of adsorption sites and significantly improved adsorption performance and recyclability. Optimal adsorption conditions were established by varying factors such as adsorbent dosage, pH, contact time, and initial dye concentration. Adsorption isotherms and kinetics conformed well to the Freundlich model and pseudo-second-order kinetic model, respectively. Thermodynamic analysis indicated spontaneous and endothermic adsorption. Mechanistically, the interaction between the modified sphagnum moss adsorbent and methylene blue involved electrostatic attraction, hydrogen bonding, and π-π interactions. In summary, modified sphagnum moss offers an efficient, cost-effective, environmentally friendly, and reusable solution for methylene blue removal from wastewater.

## Figures and Tables

**Table 1:** Langmuir and Freundlich isothermal adsorption model parameters under different solution concentrations.

Adsorption Isotherm	Parameter	298 K	308 K	318 K
Langmuir	$q_{m}$/(mg·$g^{-1}$)	195.3125	184.5018	221.3142
	$K_{L}$/(L·m$g^{-1}$)	0.0381	0.0432	0.0535
	R^2^	0.9341	0.9551	0.8999
Freundlich	$K_{F}$($mg\cdot g^{-1}$)${\left(\frac{L}{mg}\right)}^{\frac{1}{n}}$	14.2116	16.3634	17.5152
	$\frac{1}{n}$	0.5891	0.5453	0.6182
	R^2^	0.9344	0.9711	0.9181

**Table 2:** Adsorption kinetic parameters for adsorption of MB onto the modified sphagnum moss.

$q_{e}\;(exp.)$ **(** $mg\cdot g^{-1}$ **)**		**Concentration (** $mg\cdot L^{-1}$ **)**	$q_{e}\;(cal.)$ **(** $mg\cdot g^{-1}$ **)**	***k*_1_ (min^−1^)**	**R^2^**
88.76748	Pseudo-first-order	150	12.8689	0.0278	0.8453
116.89756		200	21.6427	0.0180	0.7285
		**Concentration (** $mg\cdot L^{-1}$ **)**	$q_{e}(cal.)$ **(** $mg\cdot g^{-1}$ **)**	***k*_2_ (g·mg^−1^·min^−1^)**	**R^2^**
	Pseudo-second-order	150	90.9090	0.0047	0.9988
		200	116.2791	0.0029	0.9998
		**Concentration (** $mg\cdot L^{-1}$ **)**	$k_{id}$ **(** $mg\cdot g^{-1}\cdot {min}^{-\frac{1}{2}}$ **)**	**C (** $mg\cdot g^{-1}$ **)**	**R^2^**
	Intra-particle diffusion model	150	1.6260	71.3677	0.6361
		200	1.9789	92.4940	0.6041

**Table 3:** Thermodynamic parameters of modified sphagnum moss.

$\Delta H^{0}\;(\mathrm{kJ}\cdot {mol}^{-1})$	$\Delta S^{0}\;(J\cdot {mol}^{-1}K^{-1})$	$\Delta G^{0}\;(J\cdot {mol}^{-1})$		
		298 K	308 K	318 K
94.3884	359.4830	−4080.5043	−4700.6398	−5358.9559

## Data Availability

The original contributions presented in the study are included in the article, further inquiries can be directed to the corresponding author.
